# Clinical Characteristics and Early Visual Outcomes of Highly Myopic Cataract Eyes: The Shanghai High Myopia Study

**DOI:** 10.3389/fmed.2021.671521

**Published:** 2022-01-04

**Authors:** Wenwen He, Yunqian Yao, Keke Zhang, Yu Du, Jiao Qi, Yinglei Zhang, Shaohua Zhang, Zhennan Zhao, Lei Cai, Qi Fan, Yongxiang Jiang, Jin Yang, Xiangjia Zhu, Yi Lu

**Affiliations:** ^1^Department of Ophthalmology, Eye Institute, Eye & ENT Hospital, Fudan University, Shanghai, China; ^2^NHC Key Laboratory of Myopia (Fudan University), Shanghai, China; ^3^Key Laboratory of Myopia, Chinese Academy of Medical Sciences, Shanghai, China; ^4^Shanghai Key Laboratory of Visual Impairment and Restoration, Shanghai, China

**Keywords:** highly myopic cataract, axial length, ocular characteristics, visual outcomes, risk factors

## Abstract

**Purpose:** To report ocular characteristics and early visual outcomes of highly myopic cataract eyes, and to analyze the risk factors of low vision.

**Methods:** A total of 2,027 eyes of 1,400 cataract patients with axial length (AL) ≥ 26 mm undergoing cataract surgery in Eye & ENT Hospital of Fudan University, who were registered in the Shanghai High Myopia Study, were analyzed. Routine pre-operative ophthalmic examinations were performed and macular scan of optical coherence tomography (OCT) were obtained. Macular complications, central foveal thickness (CFT) and subfoveal choroidal thickness (SFCT) were evaluated from OCT images. Ocular and surgical history and perioperative complications were also recorded. Uncorrected and best-corrected visual acuity (UCVA/BCVA) 1 month post-operatively and its influencing factors were evaluated.

**Results:** The average AL of all involved eyes was 29.52 ± 2.26 mm, and 39.7% of which were with an AL > 30 mm and 26.4% of which were with a corneal astigmatism more than 1.5 D. Nuclear cataract accounted for the largest proportion (70.6%). The rate of overall macular complications was 27.6%. Postoperative UCVA and BCVA were 0.70 ± 0.46 and 0.25 ± 0.32 logMAR, respectively. BCVA improved significantly after surgery (vs. *P* < 0.001) and affected by the elongation of AL (*P* < 0.001) and thinning of CFT and SFCT (both *P* < 0.001). The risk factors of post-operative low vision (BCVA < 20/66) were macular atrophy, lamellar macular hole, high corneal astigmatism, long AL, thin SFCT and junior surgeons, odds ratios ranging from 1.54 to 54.87 (all *P* < 0.05).

**Conclusion:** Cataract surgery could improve the VA of highly myopic eyes. Eye with macular complications, higher corneal astigmatism, longer AL, thinner SFCT, and who was treated by a junior surgeon, may have a high risk of low vision after surgery.

## Introduction

High myopia is defined as myopia ≤ −6.00 diopters (D) or axial length (AL) ≥ 26 mm (1). The incidence of high myopia is increasing rapidly worldwide (2), especially in Asian areas (3, 4). At present, there are more than 500 million myopia patients in China alone, and nearly 100 million high myopia patients (5). Correspondingly, as the most common complication of high myopia, the incidence of highly myopic cataract is increasing year by year, and it accounts for 30% of total cataract surgeries in tertiary hospitals (6).

Highly myopic cataract surgery is relatively complex and very challenging for ophthalmologists because of its association with poor fundus conditions and the difficulty of estimating the visual outcome (7). Epidemiology shows that high myopia is related to education level (8), and it tends to develop cataract almost 10 years earlier than normal eyes (6, 9). Thus, these patients usually have high expectations for visual outcome of cataract surgery. However, the uncertainty of prognosis may lead to the contradiction between doctors and patients.

Modern cataract surgery has been proved to be safe and effective in treating highly myopic cataract (9–11). However, the previous studies were usually retrospective study with a small sample size (9, 11), which provided relatively weak clinical evidence. The Shanghai High Myopia Study is a hospital-based prospective cohort study, which continuously includes highly myopic and control patients scheduled for cataract surgery at the Eye & ENT Hospital of Fudan University since October 2015. All subjects underwent detailed pre-operative examinations and prospective follow-ups (7, 12–14). The purpose of this study is to summarize the pre-operative clinical characteristics and early visual outcomes of cataract surgery in a large amount of highly myopic cataract patients from the Shanghai High Myopia Study, and to analyze the risk factors of post-operative low vision. Our study aims to provide basis for treatment of this kind of patients, especially to help surgeons predict the risk of post-operative low vision before surgery, so as to be able to fully communicate with patients in the future.

## Methods

For this study, patients underwent cataract surgery with AL ≥ 26 mm of both eyes or the operated eye were included; excluded were those whose eyes had previous trauma, corneal transplantation, uveitis, diabetic retinopathy and other ocular conditions which could affect visual acuity except high myopia and its complications or previous refractive surgery history. Patients lost to follow-up were excluded from analysis. The Institutional Review Board of the Eye & ENT Hospital of Fudan University approved the protocol of the study, and it was registered at www.clinicaltrials.gov (accession number NCT03062085). All procedures adhered to the tenets of the Declaration of Helsinki, and informed consent was obtained from each patient before registration.

### Pre-operative Examinations

Pre-operative examinations included the routine assessment of Snellen visual acuity, slit-lamp biomicroscopy, fundoscopy, non-contact tonometry (TX-20, Canon Inc., Japan), biomeasurement (IOLMaster 500 or IOLMaster 700, Carl Zeiss AG, Oberkochen, Germany), corneal topography (Pentacam HR, Oculus Inc., Wetzlar, Germany), B scans, and macular scan of optical coherence tomography (OCT, Zeiss Cirrus HD-OCT 5000; Carl Zeiss AG, Oberkochen, Germany). The following data were collected: age, gender, operative eye, visual acuity, intraocular pressure (IOP), AL, corneal curvature, cataract type (lens opacities classification system III, LOCS III grades), ocular and surgical history of the enrolled eye and systemic condition.

### Macular Complications Evaluation

Macular complications, including foveal and extrafoveal retinalschisis (RS), epiretinal membrane (ERM), lamellar and full-thickness macular hole (LMH and FMH), choroidal neovascularization (CNV) or macular atrophy, were evaluated using OCT pre-operatively. Central foveal thickness (CFT) and subfoveal choroidal thickness (SFCT) were also measured and recorded by the same doctor (Dr. W.W.H).

### Surgical Procedures

All patients underwent cataract extraction with or without intraocular lens (IOL) implantation. For the patients with IOL implantation, a foldable IOL was implanted in the capsular bag. Intraoperative complications were recorded, such as post capsular rupture. The surgeon who performed the surgery was also recorded and a surgeon with no more than 5 years of surgical training was considered as a junior surgeon.

### Post-operative Follow-Up

Patients received 1 month follow-up after surgery. Postoperative examinations included assessment of Snellen visual acuity, manifest refraction, non-contact tonometry, fundoscopy, retinal photography (Optos-200Tx, Optos, Dunfermline, UK), Macular Integrity Assessment (MAIA) microperimeter system (Ceentervue, Padova, Italy), and an OCT macular scan (Zeiss Cirrus HD-OCT 5000; Carl Zeiss AG, Oberkochen, Germany). The outcome measures of interest were post-operative visual acuity and its influencing factors.

### Statistical Analysis

Continuous variables were presented as the mean ± standard deviation (SD). Categorical variables were presented with numbers and percentages. Snellen visual acuity measurements were converted to logarithm of the minimum angle of resolution (logMAR) for statistical analyses, with counting fingers, hand motions and light perception corresponding to 1.98, 2.28, and 2.68, respectively. Because ocular characteristics and visual acuity is eye specific, analyses were run according to eye rather than patients. These were performed with all eyes combined by the generalized estimating equation method, which allows data from both eyes to be used while accounting for the correlation between the two eyes of a single patient, and analyses were adjusted for age and gender, unless otherwise stated. *Post-hoc* least significant difference (LSD) test was performed for multiple comparisons. Comparisons between pre-operative and post-operative visual acuity were performed using paired *t*-test. The relationships between post-operative visual acuity and pre-operative characters were assessed with Pearson's correlation or Spearman's correlation. Post-operative best corrected visual acuity (BCVA) <20/66 was defined as low vision, the risk factors for low vision were assessed with logistic regression. All analyses were conducted using SPSS software (version 23.0, IBM Inc.). A *P*-value of < 0.05 was considered as statistically significant.

## Results

### Baseline Characteristics

A total of 2,098 eyes from 1,448 patients were eligible and enrolled in this study. Forty-eight of these patients lost to follow-up. Finally, 2,027 eyes from 1,400 patients were able for analysis, including 601 males and 799 females.

Baseline characteristics and relevant ocular history of the included patients are summarized in Table 1. The average AL of the involved subjects was 29.52 ± 2.26 mm. Extremely high myopia (AL > 30 mm) accounted for 39.7% of all, and the maximum AL was 37.11 mm. Of these eyes, 535 (26.4%) had a corneal astigmatism more than 1.5 D, more of which were with-the-rule astigmatism. Among all the eyes, nuclear cataract accounted for the largest proportion (70.6%). In addition, previous refractive surgery occurred in 26 eyes (1.2%), while prior retinal detachment occurred in 59 eyes (2.9%).

**Table 1:** Baseline characteristics of highly myopic cataract patients.

**Baseline characteristic**	
Total eyes	2,027
Total patients	1,400
**Age (years)**	
Mean ± SD	61.48 ± 9.68
Range	18-88
**Gender**	
Males	601
Females	799
**Operated eye**	
Right	1,040
Left	987
**AL (mm)**	
Mean ± SD	29.52 ± 2.26
Range	26.00–37.11
26–28 mm	633 (31.2%)
28–30 mm	590 (29.1%)
30–32 mm	498 (24.6%)
>32 mm	306 (15.1%)
**Corneal astigmatism (D)**	
Mean ± SD	1.14 ± 0.77
Range	0.00–6.61
With-the-rule	942 (46.5%)
Against-the-rule	655 (32.3%)
0.0–1.5 D	1,492 (73.6%)
1.5–3.0 D	484 (23.9%)
>3.0 D	51 (2.5%)
**IOP (mmHg)**	
Mean ± SD	15.27 ± 3.42
Range	7.0–44.0
**Cataract type**	
Cortical	593 (29.3%)
Nuclear	1,431 (70.6%)
Posterior subcapsular	565 (27.9%)
**Ocular and surgical history**	
Glaucoma	34 (1.7%)
Prior retinal detachment	59 (2.9%)
Prior corneal laser surgery	17 (0.8%)
Prior ICL implantation	9 (0.4%)
**Systemic condition**	
High blood pressure	338 (24.1%)
Diabetes	91 (6.5%)

*SD, standard deviation; AL, axial length; D, dioper; IOP, intraocualr pressure; ICL, implantable collamer lens*.

### Macular Complications in Highly Myopic Cataract Eyes

The rate of overall macular complications was 27.6% (560/2,027), and these complications were from high myopia, not from cataract surgery, because the OCT images were all obtained before surgery. ERM was the most common macular complication in highly myopic eyes, which occurred in 329 eyes (16.2%), followed by retinalschisis (185/2,027, 9.1%). The incidences of CNV or CNV related macular atrophy and macular hole were 8.7% (177/2,027) and 4.5% (91/2,027), respectively. The average CFT and SFCT were 226 ± 62 and 85 ± 63 μm, respectively.

We then divided all the subjects into four subgroups according to AL (AL: 26–28, 28–30, 30–32, >32 mm). The incidences of macular complications in each subgroup were summarized in Table 2. CFT and SFCT decreased significantly with the elongation of AL. SFCT had a more obvious decrease than CFT, as there were significant differences in SFCT between all paired subgroups (all *P* < 0.01), while no significant differences in CFT between the two subgroups with AL ≤ 30 mm and between the two subgroups with AL > 30 mm (both *P* > 0.05). The risk of overall macular complications increased significantly with the elongation of AL (all *P* < 0.001). Each different type of macular complications had the similar change with the overall rate according to AL except full-thickness macular hole, which were rare in all subgroups.

**Table 2:** Incidence of macular complications in highly myopic cataract eyes according to axial length.

**Groups of AL**	**26–28 mm**	**28–30 mm**	**30–32 mm**	**>32 mm**
mean AL (mm)	27.14 ± 0.76	29.02 ± 0.80	30.85 ± 0.81	33.24 ± 1.17
mean CFT (μm)	235 ± 49	242 ± 64	209 ± 61	208 ± 68
mean SFCT (μm)	138 ± 70	83 ± 56	55 ± 34	41 ± 20
No complications	542/633 eyes (85.6%)	431/590 eyes (73.1%)	317/498 eyes (63.6%)	177/306 eyes (57.8%)
With at least 1 complication	91/633 eyes (14.4%)	159/590 eyes (26.9%)	181/498 eyes (36.4%)	129/306 eyes (43.2%)
**Macular complications**				
OR (95% CI)	**1 –**	**2.09** **(1.56–2.81)**	**3.50 (2.60–4.75)**	**5.45** **(3.79–7.85)**
ERM	64/633 eyes (10.1%)	85/590 eyes (14.4%)	106/498 eyes (21.3%)	74/306 eyes (24.2%)
OR (95% CI)	**1 –**	**1.43** **(1.01–2.02)**	**2.48 (1.76–3.51)**	**3.16** **(2.13–4.68)**
Foveal RS	10/633 eyes (1.6%)	22/590 eyes (3.7%)	22/498 eyes (4.4%)	14/306 eyes (4.6%)
OR (95%CI)	**1 –**	**2.31** **(1.06–5.06)**	**3.04 (1.44–6.42)**	**3.97** **(1.77–8.89)**
Extrafoveal RS	9/633 eyes (1.4%)	42/590 eyes (7.1%)	48/498 eyes (9.6%)	18/306 eyes (5.8%)
OR (95% CI)	**1 –**	**4.64** **(2.33–9.23)**	**6.74 (3.34–13.62)**	**5.98** **(2.71–13.22)**
CNV (including CNV-related macular atrophy)	22/633 eyes (3.4%)	44/590 eyes (7.5%)	61/498 eyes (12.2%)	50/306 eyes (16.3%)
OR (95% CI)	**1 –**	**2.41** **(1.48–3.94)**	**4.21 (2.49–7.11)**	**6.91** **(3.80–12.57)**
LMH	16/633 eyes (2.5%)	24/590 eyes (4.1%)	27/498 eyes (5.4%)	15/306 eyes (4.9%)
OR (95% CI)	**1 –**	**1.55** **(0.82–2.95)**	**2.14 (1.14–4.02)**	**2.26** **(1.07–4.76)**
FMH	0/633 eyes (0%)	4/590 eyes (0.7%)	2/498 eyes (0.4%)	3/306 eyes (1.0%)
OR (95%CI)	**–**	**–**	**–**	**–**

*AL, axial length; CFT, central foveal thickness; SFCT, subfoveal choroidal thickness; OR, odds ratio; CI, confidence interval; ERM, epiretinal membrane; RS, retinalschisis; CNV, choroidal neovascularization; LMH, lamella macular hole; FMH, full-thickness macular hole*.

### Summary of Cataract Surgery in Highly Myopic Cataract Eyes

All of the involved subjects completed cataract surgery. Of these eyes, 2,016 eyes underwent phacoemulsification and the other 11 eyes received extracapsular cataract extraction due to nuclear opalescence grade no <6 according to LOCSIII system. Types of IOL implanted were summarized in Table 3. Monofocal IOLs were the mostly used. Negative power IOL were used in 37 eyes (1.8%). Posterior capsular rupture (PCR) occurred in nineteen eyes (0.9%). There were 16 eyes without IOL implantation. Twelve of them were due to plano power need and with zonular weakness and poor fundus, and the other 4 were due to PCR.

**Table 3:** IOL used in highly myopic cataract eyes.

**IOL types**	**Number of eyes (%)**
**Monofocal IOL**	**1,951 (96.3%)**
Rayner 920H/970C	898 (44.3%)
MC X11 ASP	883 (43.6%)
AT LISA 409MP	96 (4.7%)
ZCB00	45 (2.2%)
SN60WF	6 (0.3%)
HOYA	19 (0.9%)
PC 525W Ergomax	4 (0.2%)
**Monofocal toric IOL**	**46 (2.3%)**
SN6ATX	14 (0.7%)
AT LISA 709M	32 (1.6%)
**Multifocal IOL**	**12 (0.6%)**
ZMB00	8 (0.4%)
AT LISA 839MP	4 (0.2%)
**Multifocal toric IOL**	**2 (0.1%)**
AT LISA 909M	2 (0.1%)
**Without IOL**	**16 (0.8%)**

### Visual Outcomes of Highly Myopic Cataract Eyes

**Figure 1:**
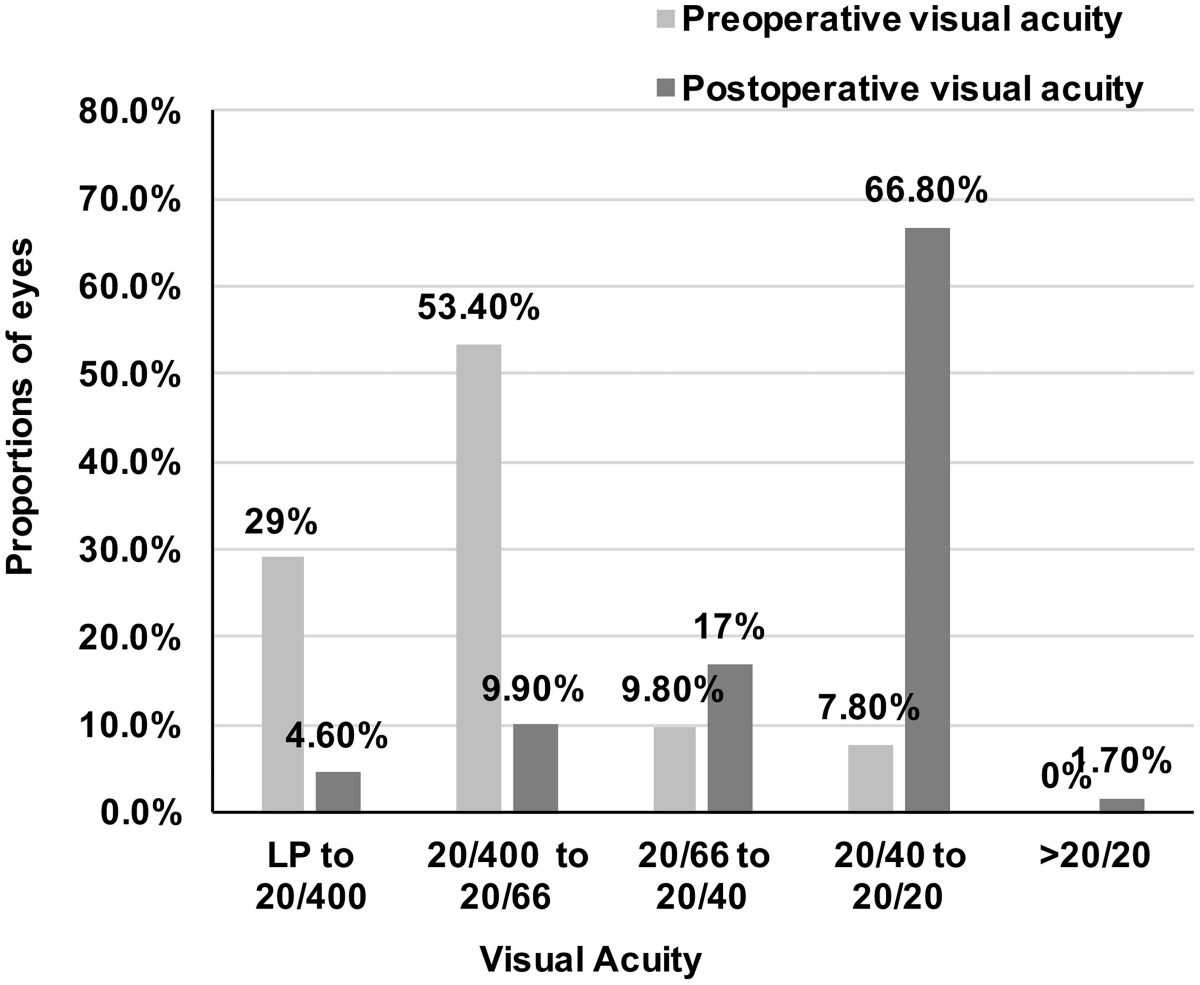
Proportions of eyes with different visual acuity before and after surgery. Visual acuity of all patients significantly improved and most patients got visual acuity between 20/40 and 20/20 (1,354/2,027 eyes, 66.8%) after cataract surgery. LP, Light perception.

The mean post-operative UCVA and BCVA were 0.70 ± 0.46 and 0.25 ± 0.32 logMAR, respectively, and both improved significantly than pre-operative visual acuity (0.95 ± 0.56 logMAR). Proportions of eyes with different visual acuity before and after surgery are shown in Figure 1. Most patients got visual acuity between 20/40 and 20/20 (1,354/2,027 eyes, 66.8%) after cataract surgery. Fifteen eyes (0.7%) developed retinal tear at 1 month follow-up with laser barrier done, one of which had PCR during surgery, and no eye developed retinal detachment during follow-up.

### Factors Influencing Post-operative VA in Highly Myopic Cataract Eyes

Table 4 lists the post-operative VA and refraction of each AL subgroup. The subgroup with AL > 32 mm had significantly worse post-operative UCVA than other subgroups (all *P* < 0.05), while post-operative BCVA became worse gradually in each subgroup (all *P* < 0.001). Postoperative myopia diopters were less in the subgroups of eyes with AL > 30 mm than the subgroups of eyes with AL ≤ 30 mm.

**Table 4:** Visual and refraction outcomes of highly myopic cataract eyes in different subgroups of axial length.

**Groups of AL**	**1: 26–28 mm**	**2: 28–30 mm**	**3: 30–32 mm**	**4: >32 mm**
Pre-operative VA	0.78 ± 0.49	0.93 ± 0.53	1.02 ± 0.54	1.26 ± 0.61
Postoperative UCVA*	0.68 ± 0.48	0.70 ± 0.44	0.68 ± 0.44	0.75 ± 0.47
Postoperative BCVA^†^	0.17 ± 0.26	0.23 ± 0.30	0.30 ± 0.35	0.38 ± 0.37
Postoperative SE (D)^#^	−3.08 ± 1.54	−3.10 ± 1.54	−2.90 ± 1.34	−2.62 ± 2.56

*VA, visual acuity; UCVA, uncorrected visual acuity; BCVA, best corrected visual acuity; SE, spherical equivalent; D, diopter*.

* *Significant difference was found between subgroup with AL > 32 mm and other subgroups (Generalized estimating equation method with post-hoc least significant difference test, P = 0.005, 0.036, and 0.001, respectively)*.

† *Post-operative BCVA became worse gradually in each subgroup (Generalized estimating equation method with post-hoc least significant difference test, all P < 0.001)*.

# *Post-operative myopia diopters were less in the subgroups of eyes with AL > 30 mm than the subgroups of eyes with AL ≤ 30 mm (Generalized estimating equation method with post-hoc least significant difference test, Subgroup 1 vs. Subgroup 3, P = 0.023, Subgroup 1 vs. Subgroup 4, P = 0.004, Subgroup 2 vs. Subgroup 3, P = 0.037, Subgroup 2 vs. Subgroup 4, P = 0.007*.

Among the 535 eyes with pre-operative corneal astigmatism more than 1.5 D, the average post-operative UCVA of the eyes with toric IOL implantation was significantly better than those eyes without toric IOL implantation after adjusting for age, gender and post-operative SE (0.52 ± 0.31 vs. 0.71 ± 0.48, *P* = 0.001). All the patients with toric IOL implantation did not require a second re-rotation surgery.

Eyes with macular complications had worse post-operative BCVA than those without (0.49 ± 0.44 vs. 0.16 ± 0.20, *P* < 0.001). Both CFT and SFCT had a negative correlation with the logMAR value of post-operative BCVA (*r* = −0.331, *P* < 0.001 and *r* = −0.317, *P* < 0.001).

### Risk Factors for Post-operative Low Vision in Highly Myopic Cataract Eyes

Table 5 shows the logistic regression model of post-operative low vision (BCVA <20/66) using generalized estimating equation method. CNV or CNV-related macular atrophy was the most important risk factor (OR = 54.87), followed by LMH, junior surgeons, higher corneal astigmatism, longer AL, and thinner SFCT.

**Table 5:** Logistic regression model of post-operative low vision in highly myopic cataract eyes.

**Parameters**	**OR**	**95% CI**	***P*** **-value**
Age	1.01	0.99–1.04	0.438
Gender	0.993	0.65–1.51	0.974
Eye	0.671	0.44–1.03	0.065
AL	1.16	1.06–1.27	0.002
Corneal astigmatism	1.54	1.20–1.98	0.001
ERM	1.68	0.97–2.94	0.066
Foveal RS	1.83	0.75–4.43	0.183
Extrafoveal RS	1.48	0.74–3.99	0.271
CNV or CNV related maculor atrophy	54.87	33.36–90.23	<0.001
LMH	3.03	1.35–6.77	0.007
FMH	27.95	0.86–911.5	0.061
CFT	0.997	0.987–1.007	0.583
SFCT	0.987	0.978–0.996	0.003
RD history	2.3	0.70–7.61	0.171
**Surgeons**			
Expert	1	–	–
Senior	1.008	0.63–1.49	0.881
Junior	2.625	1.17–5.91	0.02

*OR, odds ratio; AL, axial length; ERM, epiretinal membrane; RS, retinalschisis; CNV, choroidal neovascularization; LMH, lamella macular hole; FMH, full-thickness macular hole; CFT, central foveal thickness; SFCT, subfoveal choroidal thickness; RD, retinal detachment*.

## Discussion

Based on the epidemiology data, at least 500 million people worldwide are estimated to have high myopia, and this number would increase to one billion by 2,050 (2). In Asia area, the incidence of high myopia is much higher than elsewhere, from 2.6 to 9.1% (15, 16). Consequently, the prevalence of highly myopic cataract has also increased rapidly. However, because of the complexity of fundus conditions, it is very difficult to estimate the post-operative visual outcomes in these eyes. Therefore, to provide the evidence for clinic, we evaluated the clinical characteristics and 1-month post-operative outcomes of highly myopic cataract eyes in a large sample of 2,027 eyes from 1,400 patients. We found that cataract surgery could improve the visual acuity of highly myopic patients but the effect decreased with the elongation of axial length. Toric IOL may improve post-operative visual acuity of highly myopic cataract patients with over 1.5 D corneal astigmatism. Low vision with BCVA <20/66 was found in 14.5% of all the eyes, and the risk factors of these eyes were CNV or CNV-related macular atrophy, LMH, junior surgeons, higher corneal astigmatism, longer AL, and thinner SFCT.

Our data show that modern cataract extraction with or without IOL implantation can be safe and effective in treating highly myopic cataract patients. Both UCVA and BCVA improved in all patients and the average post-operative BCVA was comparable with previous studies (9, 17). However, our study showed a better visual outcome in extremely high myopic eyes with AL > 30 mm than the previous study (0.55 ± 0.54 logMAR) (11), probably due to the short follow-up time. Previous studies reported high axial myopia as a risk factor for pseduophakic retinal detachment (18, 19). Although there was no retinal detachment in our 1 month follow-up, there were still 15 cases occurring new retinal tear, which may because that the poor retinal was pulled by the disturbance of the vitreous body caused by the perfusion fluid during cataract surgery. Thus, regular fundal examination follow-up is particularly important for cataract patients with high myopia, which can avoid the occurrence of serious retinal complications.

However, with the extension of AL, visual outcome became worse, especially the BCVA. This may be due to the significant increase of vision-threatening macular complications such us CNV with the elongation of AL, as well as we proved before. Besides, we also found SFCT decreased significantly with the elongation of axial length rather than CFT, indicating that choroidal atrophy was more significant than retinal atrophy with the extension of axial length in patients with high myopia. Previous studies also had proved that retinal sensitivity and visual acuity were directly correlated with SFCT and did not seem to be associated with CFT in highly myopic patients (20, 21), which were similar to our results of multiple analysis. This may be because in some cases of retinal atrophy combined with ERM, the CFT is thickened, but the SFCT is still thinner. Thus, along with longer AL, thinner SFCT could be a sensitive predictor of worse vision of highly myopic cataract patients. In addition, with the elongation of axial length, post-operative myopia diopters became less, which indicated that the hyperopia drift increased, since the target refraction was more myopia for eyes with longer axial length, which was consistent with our previous results (22, 23).

Interestingly, we found corneal astigmatism affected both UCVA and BCVA after cataract surgery in highly myopic eyes. Implantation of Toric IOL in eyes with over 1.5 D corneal astigmatism improved UCVA significantly than the non-toric monofocal IOL. However, we also found high myopia as a risk factor of rotational stability of toric IOL in previous research (24). Therefore, whether a toric IOL should be used for high myopia needs to weigh the degree of corneal astigmatism and capsular stability. According to previous studies, capsular tension ring and more stable IOL type such as plate-haptic toric IOL were recommended (25–27). At the same time, the high residual astigmatism is difficult to be completely corrected by glasses and may affect the BCVA and visual function.

In addition, we found that CNV or CNV related maculor atrophy and LMH were the macular complications significantly associated with low vision after highly myopic cataract surgery. CNV or CNV related maculor atrophy is the most serious macular complication and affects VA most. Compared to LMH, FMH should have more threatening effect on VA. We also found an OR as high as 27.95 of FMH to low vision. However, the patients we included simply underwent cataract surgery, and the patients with FMH often needed combined cataract and retinal surgery, so the eyes with FMH were rare in our study and there was no statistical significance in the multivariate analysis model.

Cataract surgery underwent by junior doctors was another risk factor of post-operative low vision of highly myopic patients. A large database study showed that greater surgeon experience was associated with lower complication rates in phacoemulsification cataract extraction and with a statistically significant improvement in UCVA (28), which was similar with our results. In our study, 14 of 19 cases with intraoperative posterior capsule rupture occurred in the operation of junior doctors, which may be the cause of poor visual outcome. Therefore, it is suggested that the complicated highly myopic cataract surgery should be performed by senior doctors with certain surgical and clinical experience.

However, there were still several limitations of our study. Forty-eight patients lost to follow-up and we did not include their data in the analysis, which may add to selected basis. In addition, our follow-up time frame of this study was short and we will show long term outcomes of these patients in the future.

In conclusion, our study suggests that cataract surgery could improve the VA of highly myopic eyes. For patients with corneal astigmatism more than 1.5 D before surgery, toric IOL implantation may be needed, but caution should be taken. With the extension of axial length in highly myopic cataract eyes, the incidence of macular complications increased, and the thickness of retina and choroid became thinner, which were correlated with worse BCVA. Multiple regression analysis confirmed that CNV or CNV-related macular atrophy, LMH, high astigmatism, long AL, thin SFCT, and junior surgeons were risk factors for low vision after the surgery.

## Data Availability Statement

The raw data supporting the conclusions of this article will be made available by the authors, without undue reservation.

## Ethics Statement

The studies involving human participants were reviewed and approved by Institutional Review Board of the Eye & ENT Hospital of Fudan University, Shanghai, China. The patients/participants provided their written informed consent to participate in this study.

## Author Contributions

XZ and YL: study design and revising the manuscript. WH, YY, KZ, YD, JQ, YZ, SZ, ZZ, LC, QF, YJ, and JY: study performance. WH, YY, KZ, and YD: data collection and management. WH, YY, and KZ: data analysis and interpretation. WH: drafting the manuscript. All authors approved the manuscript.

## Funding

Publication of this article was supported by research grants from the National Natural Science Foundation of China (81970780, 81900838, 81870642, and 81670835), the Outstanding Youth Medical Talents Program of Shanghai Health and Family Planning Commission (2017YQ011), the Science and Technology Innovation Action Plan of Shanghai Science and Technology Commission (19441900700 and 21S31904900), the National Key R&D Program of China (2018YFC0116800), Clinical Research Plan of Shanghai Shenkang Hospital Development Center (SHDC2020CR4078 and SHDC12019X08), the WIT120 Research Project of Shanghai (2018ZHYL0220), Clinical Research Project of Shanghai Health and Family Planning Committee (201840199) and the Shanghai Science and Technology Commission Research Project (18ZR1435700).

## Conflict of Interest

The authors declare that the research was conducted in the absence of any commercial or financial relationships that could be construed as a potential conflict of interest.

## Publisher's Note

All claims expressed in this article are solely those of the authors and do not necessarily represent those of their affiliated organizations, or those of the publisher, the editors and the reviewers. Any product that may be evaluated in this article, or claim that may be made by its manufacturer, is not guaranteed or endorsed by the publisher.
